# Increased inflammatory cytokines in the temporal cortex in autism spectrum disorder

**DOI:** 10.1016/j.bbih.2026.101364

**Published:** 2026-09-19

**Authors:** Hadley Osman, Rachel Moreno, Elizabeth Breece, Paul Ashwood

**Affiliations:** a Department of Medical Microbiology and Immunology, University of California Davis School of Medicine, Sacramento, CA, USA; b M.I.N.D. Institute, University of California Davis, Sacramento, CA, USA

**Keywords:** Autism, Autism spectrum disorders (ASD), cytokines, IL-1, IL-18, Brain, Neuroinflammation, Superior temporal gyrus (STG), Inflammatory, Regulatory balance

## Abstract

Alterations in immune and neuroinflammatory processes are evident in neurodevelopmental disorders such as autism spectrum disorders (ASD). While prior studies using cerebrospinal fluid and plasma/serum have identified differences in cytokine levels in ASD when compared with controls, specific changes in postmortem human brain tissue remains poorly characterized. The superior temporal gyrus (STG) is involved in social communication and sensory processing, and is affected in ASD, making it a relevant region for investigating immune-related differences that has not been previously studied. Neuroimmune profiles were measured in STG tissue from 46 postmortem cases: ASD (n = 17), and neurotypical controls (n = 29). Fresh-frozen samples were collected using biopsy punches, and cytokines, chemokines, and growth factors were quantified using multiplexed bead immunoassays. In ASD cases, there were significantly increased levels of inflammatory cytokines in IL-1α, IL-8, IL-18, FLT-3L, FGF-2, G-CSF, M-CSF, MCP3, PDGF-AA and GROa compared to controls. In contrast, there was a significant decrease in RANTES, IFNα2 and IL-4 cytokine levels in ASD. Overall, ASD presented a distinctive STG cytokine profile with increased pro-inflammatory cytokine levels in the absence of an immune regulatory balance. These data highlights that unique neuroimmune pathways occur in neurodevelopmental disorders such as ASD offering insights into potential therapeutic targets.

## 1 Introduction

Autism spectrum disorders (ASD) is a common mental health condition that has increased in incidence and awareness over the last 20 years. While there is evidence for multiple genetic and environmental factors contributing to ASD, the precise etiology and pathophysiology remain unknown (Hughes et al., 2020; Hughes et al., 2018). One hypothesis is that immune dysfunction plays a significant role in the development and progression of ASD. Supporting this, findings from both epidemiological studies and animal models suggest that cytokine imbalances can disturb neurodevelopment and/or chronically impair brain function (Han et al., 2021; Hughes et al., 2023). Cytokines and their receptors are expressed by neurons and glia throughout development, influencing a range of physiological processes including neural progenitor differentiation, neuronal migration, axonal guidance, neurotrophin secretion, long-term potentiation and synaptic plasticity, which affect cognitive function, learning, and memory (Deverman and Patterson, 2009; Zengeler and Lukens, 2021). Notably, cytokines exert effects within a narrow homeostatic range and any deviations above or below physiological levels can lead to impairments in function and connectivity.

Numerous studies have examined cytokine levels in plasma, serum, and cerebrospinal fluid (CSF) to characterize immune profiles in neurodevelopmental and psychiatric disorders. Meta*-*analyses highlight elevated levels of cytokines in peripheral blood in individuals with ASD relative to healthy, typically developing, unaffected control individuals (Saghazadeh et al., 2019). Such meta-analyses of plasma revealed increased inflammatory cytokines interleukin (IL)-1β, IL-6, IL-8, interferon (IFN)γ, and monocyte chemoattractant protein (MCP)-1; however, there were lower levels of transforming growth factor (TGF)β1 (Saghazadeh et al., 2019; Masi et al., 2015). Despite these findings, there remains a knowledge gap of whether plasma and CSF cytokine levels reflect neuroinflammatory processes in the brain itself.

The superior temporal gyrus (STG) region modulates sensory integration and social emotional processes, thereby playing a critical role in integrating a breadth of information to provide meaning of the surrounding world (Jou et al., 2010). Structural and functional imaging studies have long implicated the STG in ASD (Jou et al., 2010; Amaral et al., 2008; Gros et al., 2024). Transcriptomic analyses of STG postmortem brain samples from individuals with ASD revealed neuron-specific changes in synaptic signaling, heat shock protein-related pathways, RNA splicing and upregulated AP-1-mediated signaling and insulin/IGF-1 signaling pathways compared with healthy controls (Zhang et al., 2023). Mechanistic modeling suggested a direct link between inflammation and ASD in neurons in the STG (Zhang et al., 2023). However, these gene expression studies did not identify/analyze specific genes or cytokine mediators that could take part in the neuroimmune process in the STG.

Due to the potential biological and therapeutic importance of cytokines in neurodevelopment there is a need to determine the overlap or divergence in cytokines profiles in the brain in neurodevelopmental disorders compared to controls. This study aimed to identify neuroimmune changes in individuals with ASD by quantifying levels of pro-inflammatory cytokines, chemokines and growth factors in the STG, to identify unique biomarker signatures altered in ASD compared to neurotypical controls.

## 2 Methods

### 2.1 Tissue acquisition and processing

Fresh frozen superior temporal gyrus (STG) specimens from 46 individuals, including neurotypical controls (CTRL, n = 29, 24M/5F) and autism spectrum disorder (ASD, n = 17, 14M/3F) cohorts (Supplemental Table 1) were obtained from the Autism Tissue Program (now Autism Brain Net), and M.I.N.D. Institute brain biobank at the University of California, Davis (Zhang et al., 2023). Tissue and clinical data collection procedures were approved by the institutional review board (IRB) with authorization to collect archived tissue for research. Informed consent was obtained from next-of kin at the time of brain tissue collection allowing for follow up to collect donor clinical information to confirm diagnoses and use of archived tissue for future projects. Categorical diagnoses were determined by a combination of medical records, next-of-kin (NOK) and family interviews/questionnaires, and standardized assessments, including modified versions of the Autism Diagnostic Interview-revised (ADI-R) for ASD. Controls were identified as having no recorded diagnosis of a major neurological or psychiatric disorder, and no other medical condition having a major impact on the brain. Mental health diagnoses for ASD and CTRL were confirmed by a mental health trained clinician via the Structured Clinical Interview for DSM-5 (First et al., 2015). For more information on ASD and control procedures, see: https://www.autismbrainnet.org/donor-criteria/

Whole brain specimens were bisected into separate hemispheres upon intake. One Hemispheres was coronally blocked into 1 cm thick slabs, snap frozen in liquid nitrogen vapor, and stored at −80 ^0^C until use. Samples for multiplex bead assays were collected from fresh frozen tissue blocks using 2 mm tissue biopsy punches at a depth of 2 to 3 mm from the STG, according to the anatomical definition supplied by the Atlas of the Human Brain” fourth edition (Mai et al., 2015; Zhang et al., 2023). These samples were mechanically homogenized using a Biomasher II Micro Tissue Homogenizer (Kimble) in the presence of 200ul extraction medium (0.1% Triton x, COMPLETE Protease inhibitor [Roche, 4693116001], and sterile PBS). Homogenates were centrifuged for 10 min at 2000 rpm and then the supernatant was separated into aliquots to be utilized for multiplex panels and protein normalization.

### 2.2 Cytokine measures

Brain STG concentrations of 48 cytokines and chemokines were measured using a Luminex Multiplex magnetic bead assay (Millipore Sigma,USA). 25 μl of the sample lysate was loaded onto the plate, and the assay was run according to the manufacturer's directions. The sample was incubated with antibody-conjugated fluorescent beads overnight at 4°C. Following incubation, beads were incubated with detection antibodies and then streptavidin-PE. Sample cytokine concentrations were measured using a flow-based Luminex array system (Bio-Plex 200; Bio-Rad Laboratories, Inc.) and calculated using a standard curve of known cytokine ranges. The minimum level of detection for each cytokine and chemokine are as follows (pg/mL): sCD40L: 5.65, EGF: 3.20, eotaxin: 3.08, FGF-2: 22.30, FLT-3L: 0.84, fractalkine: 29.75, G-CSF: 3.76, GM-CSF: 1.55, GROα: 1.05, IFNα2: 6.56, IFNγ: 0.86, IL-1α: 2.27, IL-1β: 0.52, IL-1RA: 1.29, IL-2: 0.28, IL-3: 0.28, IL-4: 0.20, IL-5: 0.17, IL-6: 0.14, IL-7: 0.14, IL-8: 0.52, IL-9: 3.05, IL-10: 0.91, IL-12 (p40): 3.24, IL-12 (p70): 0.88, IL-13: 2.58, IL-15: 0.74, IL-17A: 0.71, IL-17E/IL-25: 19.77, IL-17F: 28.63, IL-18: 0.53, IL-22: 12.68, IL-27: 50.78, IP-10: 2.13, MCP-1: 3.05, MCP-3: 8.61, M-CSF: 31.95, MDC: 0.42, MIG: 3.98, MIP-1α: 3.82, MIP-1β: 0.37, PDGF-AA: 10.33, PDGF-AB/BB: 16.39, RANTES: 1.58, TGFα: 0.97, TNFα: 5.39, TNFβ: 0.80, VEG-F: 0.98. A bicinchoninic acid assay (BCA; ThermoScientific, Rockford, IL) was used to determine total protein for normalization of cytokine/chemokine levels against protein variation.

### 2.3 Statistical evaluations

Descriptive statistics (frequencies, means, standard deviations, medians, quartiles) were used to summarize the cytokine/chemokine concentrations. Only analytes with a detection rate of >60% were included in analysis. Cytokines/chemokines that fell below minimum level of detection (MLD) were assigned with MLD/2. A Shapiro-Wilk test was used to assess the normality of the cytokine/chemokine data set and the test indicated non-normality of the data in the majority of analytes (*p* < 0.05). The Kruskal–Wallis rank sum test used to compare non-parametric data between groups and an ANOVA test was used for analysis parametric data, with multiple comparison correction (Sidak) used. Corrected p-values < 0.05 were considered statistically different. Data are expressed as median values (interquartile ranges). To investigate patterns between all analyzed cytokines, we created a correlation matrix using Pearson r values calculated for each pair of y values. Matrices for all cytokines in controls and all cytokines in ASD samples were made separately. We focused only on those with a Pearson r value of r > 0.8 or r < −0.8.

To evaluate the potential influence of demographic and technical covariates on cytokine concentrations, separate multiple linear regression models were fitted for each cytokine using the least-squares method. In each model, cytokine concentration served as the dependent (outcome) variable, while age (continuous), sex, and postmortem interval (PMI; continuous) were included as independent variables. Regression coefficients (β), 95% confidence intervals (CIs), *P* values, and adjusted R^2^ values were calculated for each model to assess the independent contribution of each covariate to cytokine concentration. Given the exploratory nature of these analyses and their purpose of evaluating potential confounding variables rather than testing primary biological hypotheses, each cytokine was analyzed independently.

The regression model was specified as:

Cytokine concentration = β_0_ + β_1_(Age) + β_2_(Sex) + β_3_(PMI) + ε

To determine if any of the studied cytokines were associated with an ASD diagnosis, separate multiple logistic regression models were fitted. Diagnosis was used as the outcome variable, while age and sex were included as independent variables. The regression model was specified as:

Diagnosis = β_0_ + β_1_(Age) + β_2_(Sex)

## 3 Results

Cytokine levels in the STG region differed in individuals with ASD compared to CTRL. The levels of 17 cytokines/chemokines mediators were not measured with sufficient accuracy (>60% of samples) and reproducibility above the detection limit and were excluded from further analyses, these included; GM-CSF, IFNγ, IL1β, IL-2, IL-3, IL-7, IL-12 (p40), IL-13, IL-17A, IL-17E, IL-17F, IL-22, IL-27, MIP-1α, TNFβ, PDGF AA/BB, and TNFα. Of the remaining analytes, there were unique profiles observed with significantly higher levels of IL-1α (p < 0.0001), IL-18 (p < 0.0001) FLT-3L (p < 0.0001), GROa (p = 0.0019), G-CSF (p = 0.0080), MCP-3 (p = 0.0104), IL-8 (p = 0.015), PDGF-AA (p = 0.0200), M-CSF (p = 0.046), and FGF-2 (p = 0.0469) in ASD compared with CTRL (Fig. 1, Table 1). Conversely, there were lower levels of RANTES (p = 0.0035), IFNα2 (p = 0.0065), and IL-4 (p = 0.016), in ASD compared to CTRL (Fig. 1, Table 1).

**Fig. 1:**
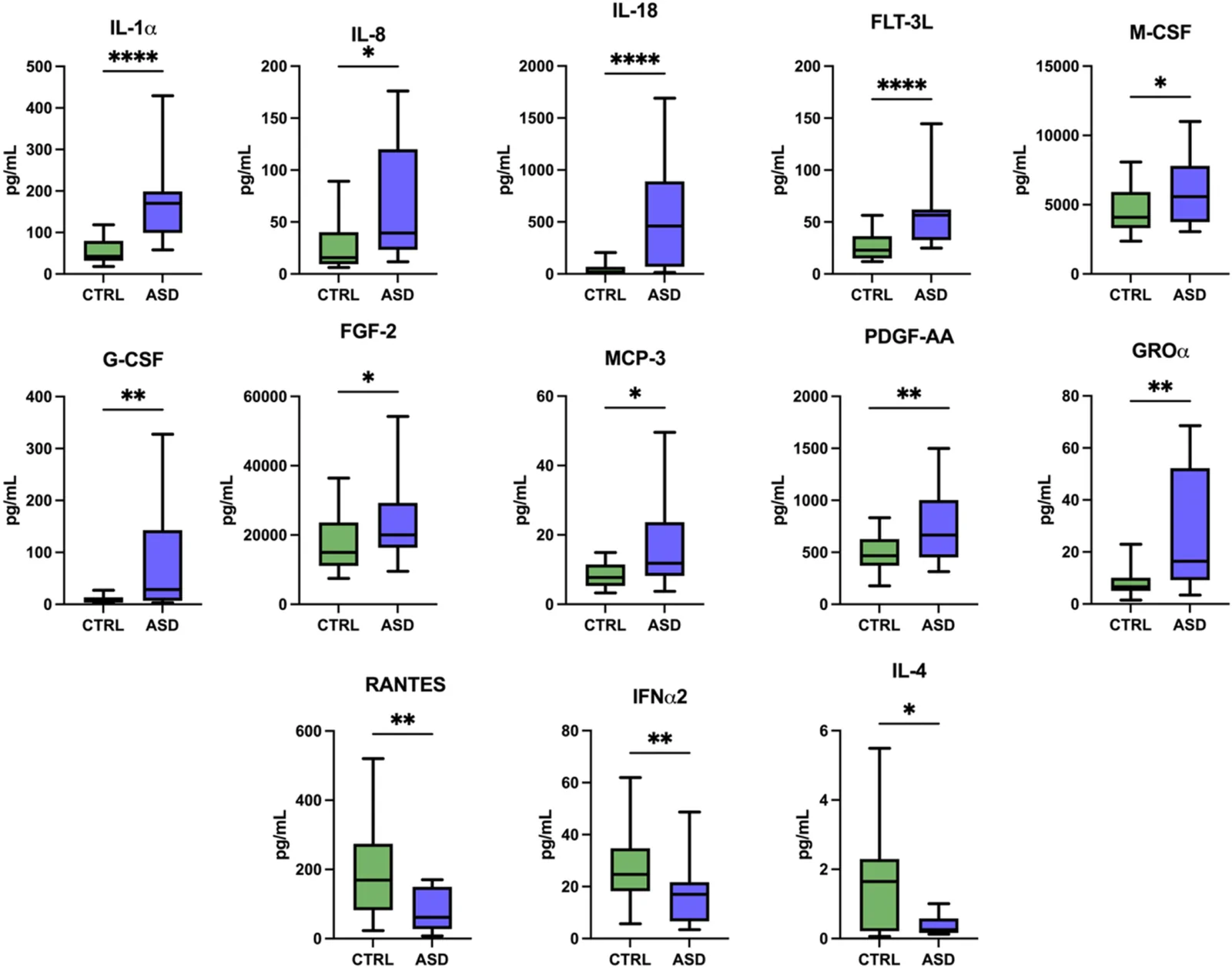
Luminex cytokine levels between control and ASD subjects. Significance determined by Mann-Whitney test. p-values are denoted as: *0.05, **0.001, ***0.0001 ****<0.0001.

**Table 1:** Differential cytokine levels in STG brain region between ASD and neurotypical controls.

Analyte		CTRL	ASD
sCD40L	Median = IQR (25th-75th) =	130.2 (66.59-350.7)	250.1 (155.6-469.9)
EGF	Median = IQR (25th-75th) =	113.1 (97.36-121.7)	104.0 (52.5-141.0)
Eotaxin	Median = IQR (25th-75th) =	76.82 (57.09-98.74)	73.83 (54.15-86.58)
FGF-2	Median = IQR (25th-75th) =	14,955* (11,091–23,644)	20,048 (16,362–29,261)
FLT-3L	Median = IQR (25th-75th) =	22.94* (14.88-36.4)	56.56 (32.52-62.11)
Fractalkine	Median = IQR (25th-75th) =	379.2 (198.1-558.1)	339.9 (257.3-631.7)
G-CSF	Median = IQR (25th-75th) =	6.329* (3.04-13.56)	28.62 (6.86-142.8)
GRO-α	Median = IQR (25th-75th) =	6.625* (5.04-10.12)	16.43 (9.14-52.26)
IFNα2	Median = IQR (25th-75th) =	24.67* (18.22-34.79)	17.06 (6.61-21.72)
IL-1α	Median = IQR (25th-75th) =	42.9* (31.72-79.91)	170.2 (98.88-199.0)
IL-1RA	Median = IQR (25th-75th) =	146.6 (104.2-201.2)	231.5 (99.63-278.6)
IL-4	Median = IQR (25th-75th) =	1.65* (0.21-2.29)	0.26 (0.16-0.58)
IL-5	Median = IQR (25th-75th) =	0.29 (0.24-0.43)	0.43 (0.24-0.61)
IL-6	Median = IQR (25th-75th) =	4.16 (0.50-40.36)	9.15 (1.91-54.5)
IL-8	Median = IQR (25th-75th) =	15.76* (9.48-40.23)	39.46 (23.19-120.1)
IL-9	Median = IQR (25th-75th) =	11.55 (9.9-13.41)	13.6 (8.29-22.64)
IL-10	Median = IQR (25th-75th) =	0.71 (0.49-1.22)	0.95 (0.69-1.45)
IL-12 p70	Median = IQR (25th-75th) =	0.83 (0.59-1.19)	1.03 (0.76-1.49)
IL-15	Median = IQR (25th-75th) =	20.57 (16.37-24.9)	22.67 (19.46-27.19)
IL-18	Median = IQR (25th-75th) =	7.86* (4.89-63.38)	461.2 (69.32-891.2)
MCP-1	Median = IQR (25th-75th) =	139.9 (97.56-199.8)	161.5 (126.8-312.6)
MCP-3	Median = IQR (25th-75th) =	7.71* (5.28-11.5)	11.83 (8.16-23.67)
M-CSF	Median = IQR (25th-75th) =	4098* (3311–5915)	5583 (3743–7812)
CXCL9	Median = IQR (25th-75th) =	48.95 (30.66-95.06)	40.92 (21.35-68.46)
MIP-1β	Median = IQR (25th-75th) =	3.6 (2.04-6.58)	1.81 (0.48-6.34)
PDGF-AA	Median = IQR (25th-75th) =	467.9* (370.2-627.7)	666.6 (450.3-1003)
RANTES	Median = IQR (25th-75th) =	169.5* (82.09-274.3)	61.54 (27.58-149.6)
TGF-α	Median = IQR (25th-75th) =	14.77 (9.21-19.08)	14.48 (6.17-22.8)
VEG-F	Median = IQR (25th-75th) =	8.54 (3.43-25.37)	8.21 (1.89-30.51)
IP-10	Median = IQR (25th-75th) =	1.25 (1.09-4.45)	22.9 (1.7-47.7)
MDC	Median = IQR (25th-75th) =	0.88 (0.57-1.59)	1.19 (0.67-1.51)

*p < 0.05 compared with ASD.

In order to compare patterns of cytokines between the control and ASD groups, correlation matrices were made with Pearson r values for each cytokine comparison. In the control group, IL-6 was strongly positively correlated with IL-8 (r = 0.801) and IL-10 (r = 0.945). MIP-1β and MCP-1 had a Pearson r value of 0.845. IP-10 was strongly correlated with IL-15 (r = 0.842) and CXCL9 (r = 0.990) (Fig. 2a). The patterns of cytokine correlations shifted in the ASD samples. While the correlation between IP-10 and CXCL9 remained strong (r = 0.992), the other strong correlations from the control samples were lost. In addition, new correlations were observed for IL-6 correlated with MCP-1 (r = 0.965), CXCL9 (r = 0.952), and IP-10 (r = 0.940). Moreover, several strong correlations with sCD40L, including with G-CSF (r = 0.863), MCP-1 (r = 0.902), and MCP-3 (r = 0.965) (Fig. 2b). No strong negative (r < −0.8) correlations were seen in the control or ASD groups.

**Fig. 2:**
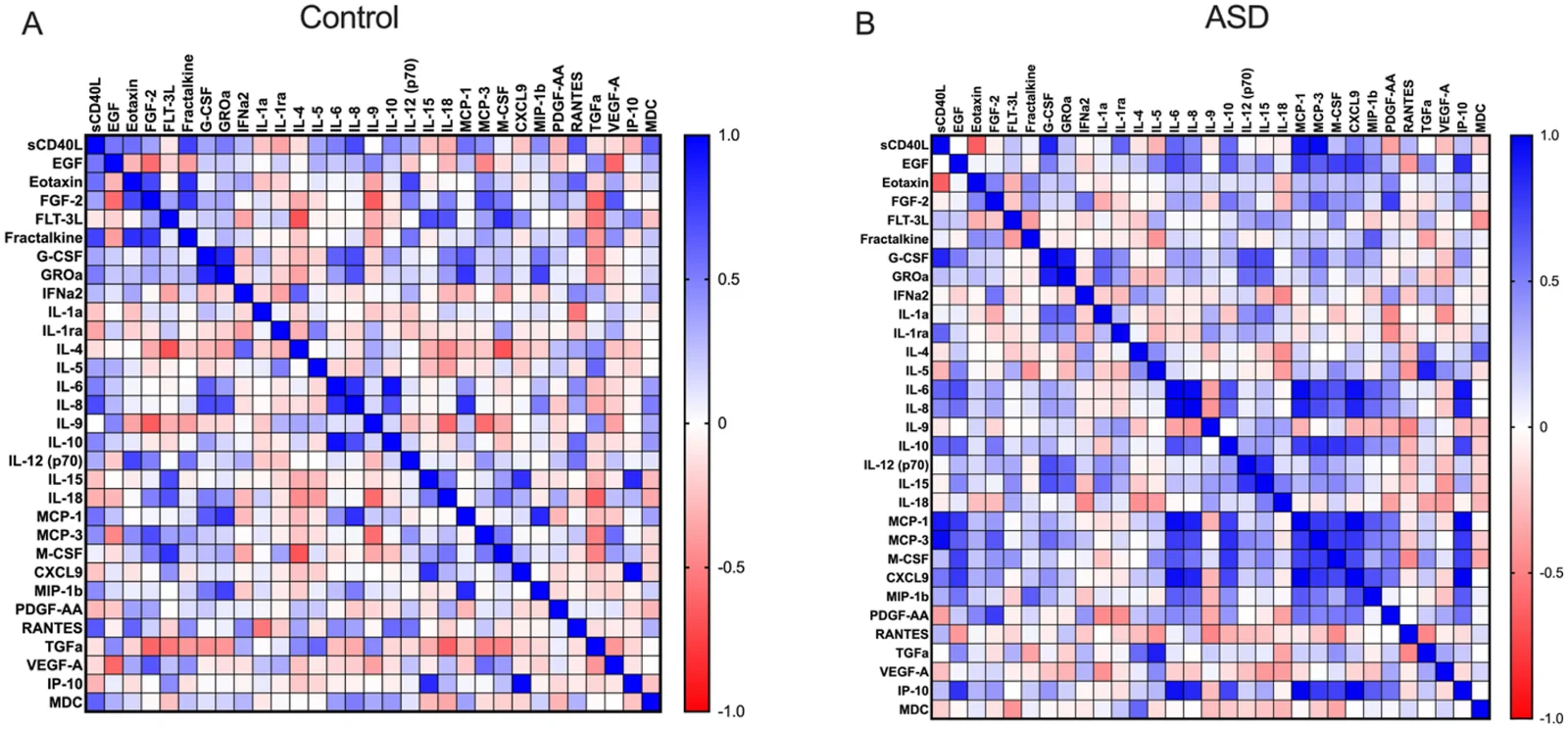
Pearson r value correlation tables between all in-range cytokines.

To evaluate potential confounding effects, separate multiple linear regression models were fitted for each cytokine including age, sex, and PMI as predictors. Overall, few cytokines demonstrated associations with age or sex. In contrast, although we did not have complete data for all subjects (Supplemental Table 1) several cytokines had associations with PMI, including IL-18, IP-10, MCP-1, TGFα, IL-5, and EGF, suggesting that tissue collection interval may contribute to variability in cytokine concentrations. Collectively, these findings indicate that PMI may represent a more important covariate than age or sex in subsequent analyses. However, the adjusted R^2^ for each analysis was small, therefore it is possible these factors are only making minor contributions to changes in cytokine concentration.

Logistic regression analysis identified several cytokines significantly associated with ASD diagnosis (Supplemental Table 2). Higher levels IL-1α, IL-1RA, IL-9, MCP-3, M-CSF, PDGF-AA, and FGF-2, were associated with increased odds of ASD, whereas higher IL-4 levels were associated with decreased odds of ASD (Supplemental Table 2). No significant associations were observed with ASD diagnosis or the remaining cytokines examined.

## 4 Discussion

Glial cells and neurons produce and respond to a diverse array of cytokines that influence their function and phenotype (Deverman et al., 2009; Zengeler et al., 2021). Current dogma suggests a tight physiological range for cytokines to maintain proper brain development and function with deviations either side of this range resulting in altered brain plasticity and behavior. Cytokines, act on neural progenitors to alter neuronal populations, migration, and brain development. They also alter synapse formation to either modulate the expression and function of synaptogenesis proteins or to activate signaling pathways that destabilize or eliminate synapses. Altered synaptic pruning and synaptic connectivity have been proposed as mechanisms for neurodevelopmental disorders (Germann et al., 2021; Van Spronsen et al., 2010; Pagani et al., 2021; Thomas et al., 2016; Cunningham et al., 2013). These mechanisms are not mutually exclusive and may act in concert or over different time courses to alter neurodevelopment. The precise physiological role of cytokines in the brain is not fully understood, but gain- and loss-of-function studies suggest these molecules can regulate key developmental processes. Our data showed unique profiles of cytokines in the STG in ASD, a region that is important in sensory and language processing. Individuals with ASD exhibited elevated levels of the IL-1 family of cytokines (IL-1α and IL-18), FLT-3L, M-CSF, G-CSF, MCP3, FGF-2, PDGF-AA, IL-8, and GROα compared to neurotypical controls. In contrast individuals with ASD did not have a compensatory change in regulatory cytokines such as IL-10 even in the presence of this increased proinflammatory profile. This lack of immune regulatory control in ASD has been described in cytokine levels in plasma an in other tissues such as the ileum and colon (Moreno et al., 2025). The impaired cytokine regulation in ASD may contribute to an immune environment dominated by net inflammatory signals.

Elevated proinflammatory cytokine levels have been reported in other brain regions of individuals with ASD, including the cerebellum, anterior cingulate gyrus and frontal cortex (Li et al., 2009; Vargas et al., 2005; Wei et al., 2012). Furthermore, multiple transcriptomic studies of postmortem brain tissue consistently highlight activation of innate immunity/glial markers and cytokine signaling pathways in ASD (Hughes et al., 2023). Among these studies, elevated IL-1 levels in the serum, CSF, and/or brains of individuals with ASD have consistently been reported (Masi et al., 2015; Vargas et al., 2005; Arenella et al., 2023; Ashwood et al., 2011a, Ashwood et al., 2011b; Hughes et al., 2022; Sreenivas et al., 2024). In addition, genetic defects in the IL-1 family of cytokines and receptors have been reported in ASD (Arenella et al., 2023; Allen-Brady et al., 2010; Piton et al., 2008). Among these are genes that encode the IL-1 receptor accessory protein-like 1 (IL1RAPL1) and IL-1 receptor accessory protein (IL-1RAcP) which are synapse-organizing proteins that mediate cell adhesion and recruitment of glutamatergic synaptic components (Choucair et al., 2015; Takahashi et al., 2013). Mice with *IL1RAPL1* deletion exhibit some ASD-like behaviors, as well as deficits in associative memory and cognition (Houbaert et al., 2013; Yasumura et al., 2014). The IL-1 family consists of 11 known members, including IL-1α, IL-1β, IL-1Ra and IL-18 (Gaballa et al., 2024). In this study both IL-1 and IL-18 were differentially increased in ASD brains. IL-1 and its receptors play important roles in neurological processes from early CNS development through adulthood, influencing neurogenesis, proliferation, migration, synapse formation and plasticity (Mantovani et al., 2019; Dinarello et al., 2018). IL-1 was the first cytokine identified with actions on the CNS, inducing fever and *de novo* production of IL-1 in the brain following peripheral injection of IL-1 (Mantovani et al., 2019; Besedovsky et al., 1986). In addition, IL-1 influences pain modulation, stress responses, and can induce behavioral changes after acute or repeated administration (Mantovani et al., 2019; Besedovsky et al., 1986; Bonaccorso et al., 2003). Elevated levels of IL-1 in the brain are linked with memory and cognitive impairments (Goshen et al., 2009; Viviani et al., 2003). Similarities between the IL-1 and IL-18 receptor complexes and signaling, and their abilities to modulate fever, stress, and behavioral responses, suggest that they mediate similar effects in ASD (Alboni et al., 2010).

Similar to our findings in STG brain tissue, increased levels of IL-8 are detected in serum, CSF, and brain tissues of individuals with ASD (Masi et al., 2015; Vargas et al., 2005; Ashwood et al., 2011a, Ashwood et al., 2011b; Singh et al., 2017; Pardo et al., 2017). Increased IL-8 levels have also been found in the CSF in depressive disorders (Runge et al., 2021, 2023; Kuzior et al., 2020). Furthermore, elevated serum IL-8 present within the first three weeks of life is predictive of poorer neurodevelopmental outcomes (Nist et al., 2019). Our data also showed that the proliferation survival factor FLT-3L and cytokine/growth factors M-CSF and G-CSF were increased in ASD brain. FLT-3L is important in dendritic cell (DC) function in the immune system and is increased in the CSF of individuals with ASD (Pardo et al., 2017). Previously, DC subsets were associated with abnormal amygdala volumes in children with ASD (Breece et al., 2013). These findings indicate activation of DC subsets in ASD may contribute to an amplified inflammatory response. Increased M-CSF can lead to microglia activation, microglia proliferation and release of IL-1 (Hao et al., 2002; Li et al., 2021). Increased M-CSF levels are negatively correlated with verbal ability in children with ASD (Rose and Ashwood, 2014). G-CSF levels are increased in plasma cord blood samples from ASD compared to unaffected sibling controls (Moreno et al., 2024) and remain elevated into early adolescence (Belica et al., 2023). G-CSF may be released due to brain injury as blocking G-CSF in maternal immune activation and gestational valproic acid exposure models rescue behaviors and reduces neuroinflammation in offspring (Durankuş et al., 2022; Mishra et al., 2022). FGF-2 promotes the proliferation of neural progenitor cells and astrocytes, influences neuronal function and brain growth, and can alter blood brain barrier (BBB) permeability (Reuss et al., 2003; van Scheltinga et al., 2010; Yoshimura et al., 2001). PDGF regulates multipotent progenitors that are distinct from neural stem cells (Kumari et al., 2020; Moore et al., 2013). MCP-3 is produced by astrocytes, acts as a differentiating factor for dopaminergic precursors and neurons as well as activate microglia following brain injury (Edman et al., 2008; Renner et al., 2011). Together, these neuro-inflammatory mediators may play diverse roles in early brain development, synaptogenesis and neuronal network reorganization and, when disrupted, may contribute to ASD.

Interestingly, IFNα2 and IL-4 levels were significantly lower in STG brain tissue of ASD. The role of IFNα2 in neuroimmune processes or ASD has not been well described. In contrast, IL-4, primarily produced by T helper-2 (T_H_2) cells, is known to be dysregulated in ASD (Ashwood et al., 2011a, Ashwood et al., 2011b; Careaga et al., 2017; Nie et al., 2023; Rose et al., 2018, 2020). IL-4 plays a crucial role in neuroprotection following injury (Walsh et al., 2015) and regulates learning and memory (Derecki et al., 2010). IL-4 can shift microglia function, skewing to a phenotype that facilitates tissue repair, alleviates neuronal damage (He et al., 2020), and dampens astrocyte activation (Brodie et al., 1998). The reduction of IL-4 in ASD suggests a diminished capacity to regulate microglial activation and astrocyte responses, contributing to a prolonged neuroinflammatory state (Vargas et al., 2005). Lower levels of IL-4 in ASD may also reflect an imbalance that favors a more neuroinflammatory environment. The chemokine, RANTES, was also decreased in ASD. RANTES has neuroinflammatory roles in the context of traumatic brain injury, but little is known about its role in neurodevelopment (Mennicken et al., 1999; Dutta et al., 2019; Bedrossian et al., 2016; Lesh et al., 2018).

Correlation data revealed patterns that we did not detect in group-to-group comparisons of individual cytokines. While some of the cytokines, such as IL-8, G-CSF, and MCP-3 were significant in our initial analysis of cytokine levels (Fig. 1), many cytokines that strongly correlated with our significant cytokines were not present in the group analysis. As cytokines can have a combinatorial effect on cells, inflammatory processes, neurodevelopment, and behaviors, using a more global approach to look at interactions provides a different viewpoint to determine changes between ASD and controls. Evaluating which cytokine levels are related to others can help in future studies to understand the effects of combined cytokine influences compared to their individual impacts on different brain cell types. We found that correlations apparent in controls were not always present in ASD, suggesting divergence from controls. Future experiments designed to investigate the net effect of cytokines that were different in ASD on neuronal function are warranted.

While this study provides valuable insights into neuroimmune differences in ASD, certain limitations should be considered. Postmortem brain studies inherently involve variability in factors such as cause of death, co-morbid conditions, PMI, and medication history. In linear regression analyzes we did not find a big effect of age and sex on cytokine levels and PMI only produced a minor influence over a few cytokines. Of the cytokines that were significantly different between ASD and controls in group analyses, only IL-18 was mildly affected by PMI. As such, the replication of the IL-18 results are recommended. We did not have data on medications, but it is possible that medication use, particularly antipsychotics, may also influence cytokine levels, but prior studies suggested these medications do not fully account for observed differences (Chen et al., 2023; Li et al., 2022; Simsek et al., 2016). Moreover, rigorous tissue processing protocols help mitigate these concerns. In addition, this study did not include direct behavioral data linked to immune markers, but future work integrating clinical datasets, such as those from Autism BrainNet (SFARI) and the NIH NeuroBiobanks, could clarify associations between cytokine profiles and symptom severity. Future studies should also include participants that have more information on social demographics, medical history, immune medical history, drugs of choices, etc. While most subjects were male, reflecting ASD prevalence, expanding female representation will be important for understanding potential sex-specific neuroimmune differences. Lastly, this novel study focused on the STG, a region implicated in both ASD, but investigating cytokine expression across multiple brain areas will provide a broader perspective on immune involvement in these conditions. Our future studies will expand on current findings to map cytokine levels to specific cell types in multiple brain regions, in relation to clinical characteristics across the human lifespan.

## 5 Conclusions

Increased cytokine and chemokine levels were seen in the STG of individuals with ASD compared to controls. ASD brains had increased inflammatory cytokine profiles but lacked a compensatory change in regulatory cytokines, suggesting a more pronounced shift towards neuroinflammation and away from homeostasis. Cytokine changes could represent detrimental, adaptive, or neutral outcomes (or all three) depending on when and where they occur in the brain. Determining the relationship between elevated cytokines and the neuropathology of ASD requires careful evaluations at a region-specific manner over a lifespan. Further investigation into the timing of these immune changes and their potential as therapeutic targeting could provide valuable insights for developing novel treatment strategies for ASD.

## CRediT authorship contribution statement

**Hadley Osman:** Writing – review & editing, Methodology, Investigation, Formal analysis. **Rachel Moreno:** Formal analysis, Writing – review & editing. **Elizabeth Breece:** Formal analysis, Data curation. **Paul Ashwood:** Writing – original draft, Supervision, Resources, Project administration, Methodology, Investigation, Funding acquisition, Formal analysis, Data curation, Conceptualization.

## Ethics declaration

At the time of tissue donation consent was provided by next of Kin for future use of tissue in research.

This study included organ or tissue donors. This study includes human biological material and consent was obtained by donors, or their next of kin or legal representatives, for use in this study. The samples used in this research were not sourced from executed prisoners or prisoners of conscience.

This study was performed in compliance with relevant laws, regulatory frameworks and guidelines where the research took place. This study was approved by the institutional review boards at the University of California, Davis. The authors provided the following explanation: Archive tissue use exemption from IRB.

## Declaration of competing interest

The authors declare no conflicts of interest.

## Data Availability

Data will be made available on request.
