# Supplementary material for: Increased inflammatory cytokines in the temporal cortex in autism spectrum disorder
Source: Brain Behav Immun Health. 2026 Sep 19;57:101364. doi: 10.1016/j.bbih.2026.101364 (PMC13634538; doi:10.1016/j.bbih.2026.101364)
Supplement: Supplementary file 1 [file mmc1.docx]

**Table 1. Case information**

| Control | | | |  | Autism Spectrum Disorder | | | | |  |
| --- | --- | --- | --- | --- | --- | --- | --- | --- | --- | --- |
| Case | Age (yr) | Sex | PMI (hr) |  | | Case | Age (yr) | Sex | PMI (hr) |  |
| Control-1 | 12 | M | 13 |  | | ASD-1 | 10 | M | - |  |
| Control-2 | 13 | F | 17 |  | | ASD-2 | 11 | F | 13 |  |
| Control-3 | 13 | M | 19 |  | | ASD-3 | 15 | M | - |  |
| Control-4 | 15 | M | 16 |  | | ASD-4 | 18 | M | - |  |
| Control-5 | 15 | M | 31 |  | | ASD-5 | 20 | M | 23 |  |
| Control-6 | 16 | M | 19 |  | | ASD-6 | 20 | M | - |  |
| Control-7 | 18 | F | 8 |  | | ASD-7 | 27 | M | 8 |  |
| Control-8 | 19 | M | 14 |  | | ASD-8 | 30 | M | 20 |  |
| Control-9 | 19 | M | 57 |  | | ASD-9 | 30 | M | 16 |  |
| Control-10 | 20 | F | 35 |  | | ASD-10 | 38 | M | - |  |
| Control-11 | 21 | M | 30 |  | | ASD-11 | 39 | M | 23 |  |
| Control-12 | 22 | M | 18 |  | | ASD-12 | 39 | M | 14 |  |
| Control-13 | 26 | M | 18 |  | | ASD-13 | 41 | F | - |  |
| Control-14 | 27 | M | 36 |  | | ASD-14 | 43 | M | - |  |
| Control-15 | 28 | M | 24 |  | | ASD-15 | 49 | F | 16 |  |
| Control-16 | 30 | M | 20 |  | | ASD-16 | 65 | M | - |  |
| Control-17 | 31 | M | 19 |  | | ASD-17 | 73 | M | - |  |
| Control-18 | 31 | F | 17 |  | |  |  |  |  |  |
| Control-19 | 32 | M | 32 |  | |  |  |  |  |  |
| Control-20 | 33 | M | 24 |  | |  |  |  |  |  |
| Control-21 | 37 | M | 34 |  | |  |  |  |  |  |
| Control-22 | 38 | M | 25 |  | |  |  |  |  |  |
| Control-23 | 39 | M | 14 |  | |  |  |  |  |  |
| Control-24 | 39 | M | 24 |  | |  |  |  |  |  |
| Control-25 | 44 | M | 23 |  | |  |  |  |  |  |
| Control-26 | 46 | M | 24 |  | |  |  |  |  |  |
| Control-27 | 46 | F | 20 |  | |  |  |  |  |  |
| Control-28 | 46 | M | 31 |  | |  |  |  |  |  |
| Control-29 | 49 | M | 31 |  | |  |  |  |  |  |
