# Supplementary material for: Increased inflammatory cytokines in the temporal cortex in autism spectrum disorder
Source: Brain Behav Immun Health. 2026 Sep 19;57:101364. doi: 10.1016/j.bbih.2026.101364 (PMC13634538; doi:10.1016/j.bbih.2026.101364)
Supplement: Supplementary file 2 [file mmc2.docx]

| Cytokine | Age β (95% CI) | *P* | Sex β (95% CI) | *P* | PMI β (95% CI) | *P* | Adjusted R² |
| --- | --- | --- | --- | --- | --- | --- | --- |
| IL-8 | -3.98 (-14.25, 6.29) | 0.435 | 160.9 (-128.4, 450.0) | 0.265 | -13.99 (-30.33, 2.34) | 0.091 | 0.132 |
| IL-9 | 0.02 (-0.14, 0.18) | 0.798 | 3.23 (-1.97, 8.41) | 0.212 | -0.18 (-0.50, 0.14) | 0.255 | -0.032 |
| IL-10 | -0.34 (-0.96, 0.28) | 0.250 | 4.26 (-16.30, 24.81) | 0.657 | 0.20 (-1.31, 1.72) | 0.773 | -0.086 |
| IL-12p70 | 0.03 (-0.001, 0.05) | 0.062 | -0.33 (-1.25, 0.59) | 0.467 | -0.02 (-0.07, 0.02) | 0.333 | 0.081 |
| IL-13 | 0.01 (-0.25, 0.28) | 0.908 | 2.24 (-6.20, 10.67) | 0.568 | -0.34 (-0.83, 0.16) | 0.162 | -0.026 |
| IL-15 | 0.90 (-0.75, 2.55) | 0.267 | 4.03 (-41.95, 50.00) | 0.857 | 0.93 (-1.14, 2.99) | 0.359 | 0.033 |
| IL-17 | 0.01 (-0.07, 0.09) | 0.832 | -0.99 (-3.34, 1.35) | 0.393 | 0.02 (-0.11, 0.15) | 0.775 | -0.069 |
| IL-18 | 1.74 (-29.38, 32.86) | 0.910 | -257.7 (-1149, 633.2) | 0.560 | **61.41 (11.30, 111.50)** | **0.018** | 0.098 |
| IP-10 | **-14.02 (-27.71, -0.32)** | **0.046** | -308.6 (-758.3, 141.2) | 0.161 | **34.65 (13.05, 56.26)** | **0.004** | 0.398 |
| MCP-1 | 7.47 (-141.1, 156.0) | 0.919 | 1121 (-3132, 5374) | 0.595 | **-249.2 (-488.4, -9.97)** | **0.042** | 0.046 |
| MCP-3 | 0.14 (-0.19, 0.47) | 0.385 | -0.15 (-10.28, 9.98) | 0.976 | -0.34 (-0.93, 0.26) | 0.256 | -0.039 |
| M-CSF | 55.17 (-32.41, 142.7) | 0.208 | 48.71 (-2459, 2556) | 0.969 | -120.5 (-261.5, 20.52) | 0.091 | 0.028 |
| MDC | -0.01 (-0.03, 0.01) | 0.464 | 0.29 (-0.37, 0.96) | 0.370 | -0.02 (-0.06, 0.01) | 0.238 | -0.004 |
| CXCL9 | 6.48 (-12.62, 25.57) | 0.494 | 160.5 (-386.1, 707.1) | 0.554 | -1.83 (-32.58, 28.91) | 0.904 | -0.065 |
| MIP-1β | 0.80 (-0.90, 2.49) | 0.342 | -45.35 (-91.84, 1.14) | 0.056 | -0.70 (-2.93, 1.52) | 0.521 | 0.063 |
| PDGF-AA | 6.37 (-2.70, 15.44) | 0.162 | -33.44 (-293.1, 226.2) | 0.795 | -8.41 (-23.01, 6.19) | 0.249 | 0.000 |
| PDGF | -9.87 (-31.45, 11.70) | 0.338 | -334.2 (-1020, 352.0) | 0.310 | -24.32 (-59.43, 10.79) | 0.157 | 0.245 |
| RANTES | -0.75 (-8.43, 6.92) | 0.843 | -58.97 (-278.6, 160.7) | 0.588 | 3.94 (-8.42, 16.29) | 0.521 | -0.076 |
| TGFα | 0.17 (-0.10, 0.44) | 0.211 | 5.15 (-2.53, 12.83) | 0.181 | **-0.50 (-0.93, -0.07)** | **0.024** | 0.109 |
| TNFα | 0.05 (-0.49, 0.59) | 0.843 | 13.11 (-4.98, 31.20) | 0.145 | -0.69 (-1.59, 0.22) | 0.128 | 0.026 |
| VEGF | -0.17 (-0.71, 0.38) | 0.541 | 7.23 (-8.39, 22.86) | 0.352 | 0.17 (-0.71, 1.05) | 0.695 | -0.047 |
| sCD40L | -3.29 (-17.70, 11.13) | 0.618 | 265.1 (-163.1, 693.3) | 0.195 | -12.68 (-29.43, 4.08) | 0.121 | 0.293 |
| FGF-2 | 90.26 (-243.2, 423.7) | 0.585 | -6346 (-15892, 3200) | 0.185 | 128.9 (-408.0, 665.9) | 0.628 | -0.026 |
| Fractalkine | 3.12 (-9.78, 16.01) | 0.625 | -85.78 (-454.9, 283.3) | 0.639 | 13.67 (-7.09, 34.43) | 0.189 | -0.016 |
| G-CSF | -7.83 (-60.60, 44.93) | 0.762 | 614.0 (-897.6, 2126) | 0.411 | -43.45 (-137.5, 50.61) | 0.351 | -0.055 |
| GROα | 0.21 (-0.87, 1.29) | 0.694 | -7.73 (-38.74, 23.27) | 0.615 | 0.01 (-1.74, 1.75) | 0.995 | -0.083 |
| IL-1α | 0.87 (-0.90, 2.64) | 0.325 | 19.15 (-31.48, 69.78) | 0.446 | -2.13 (-4.98, 0.72) | 0.138 | 0.090 |
| IL-1Ra | -4.06 (-8.99, 0.88) | 0.104 | 19.78 (-121.5, 161.0) | 0.777 | 2.11 (-5.84, 10.05) | 0.592 | -0.003 |
| IL-4 | **2.56 (0.98, 4.14)** | **0.002** | 19.19 (-35.88, 74.26) | 0.483 | 0.86 (-2.06, 3.78) | 0.554 | 0.282 |
| IL-5 | 0.006 (-0.001, 0.012) | 0.075 | 0.16 (-0.03, 0.35) | 0.090 | **-0.014 (-0.025, -0.004)** | **0.008** | 0.195 |
| IL-6 | -30.80 (-80.55, 18.95) | 0.216 | 649.4 (-775.0, 2074) | 0.360 | -28.56 (-108.7, 51.56) | 0.473 | 0.001 |
| EGF | -0.49 (-2.03, 1.05) | 0.520 | 36.80 (-12.59, 86.19) | 0.138 | **-3.80 (-6.52, -1.07)** | **0.008** | 0.202 |
| Eotaxin | **1.16 (0.01, 2.32)** | **0.049** | -5.50 (-38.56, 27.57) | 0.737 | 0.91 (-0.95, 2.77) | 0.327 | 0.091 |

**Supplemental Table 1**: Analysis of potential confounding effects. Separate multiple linear regression models were fit for each cytokine including age, sex, and postmortem interval (PMI) as predictors. Overall, few cytokines demonstrated associations with age or sex. IL-18, IP-10, MCP-1, TGFα, IL-5, and EGF had associations with PMI.

| Cytokine | Odds Ratio | 95% CI | p-value | Result |
| --- | --- | --- | --- | --- |
| IL-8 | 1.000 | 0.9983–1.003 | 0.6883 | Not significant |
| IL-9 | **1.361** | **1.101–1.892** | **0.0014** | **↑ Significant** |
| IL-10 | 0.5264 | 0.1298–1.010 | 0.0585 | ↓ Trend |
| IL-12p70 | 1.521 | 0.6327–4.112 | 0.3489 | Not significant |
| IL-13 | 1.165 | 0.8831–1.697 | 0.2695 | Not significant |
| IL-15 | 0.9913 | 0.9539–1.016 | 0.5049 | Not significant |
| IL-17 | 1.230 | 0.9592–1.615 | 0.1031 | Not significant |
| IL-18 | 1.000 | 0.9996–1.001 | 0.4425 | Not significant |
| IP-10 | 0.9985 | 0.9916–1.002 | 0.4721 | Not significant |
| MCP-1 | 1.000 | 0.9999–1.000 | 0.5034 | Not significant |
| MCP-3 | **1.097** | **1.012–1.221** | **0.0212** | **↑ Significant** |
| M-CSF | **1.000** | **1.000–1.001** | **0.0076** | **↑ Significant** |
| MDC | 1.305 | 0.7244–2.614 | 0.3576 | Not significant |
| CXCL9 | 0.9991 | 0.9942–1.001 | 0.3091 | Not significant |
| MIP-1β | 0.9912 | 0.9316–1.015 | 0.5408 | Not significant |
| PDGF-AA | **1.003** | **1.000–1.006** | **0.0269** | **↑ Significant** |
| RANTES | 0.9997 | 0.9975–1.002 | 0.7864 | Not significant |
| TGF-α | 0.9970 | 0.9269–1.071 | 0.9338 | Not significant |
| TNF-α | 1.061 | 0.9799–1.193 | 0.1409 | Not significant |
| VEGF-A | 1.014 | 0.9734–1.056 | 0.4952 | Not significant |
| sCD40L | 1.005 | 0.9995–1.013 | 0.0771 | Not significant |
| FGF-2 | **1.000** | **1.000–1.000** | **0.0387** | **↑ Significant** |
| Fractalkine | 0.9992 | 0.9970–1.001 | 0.3598 | Not significant |
| G-CSF | 1.000 | 0.9999–1.001 | 0.1265 | Not significant |
| GRO-α | 1.008 | 0.9920–1.024 | 0.3307 | Not significant |
| IL-1A | **1.056** | **0.02691–0.1036** | **<0.0001** | **↑ Significant** |
| IL-1RA | **1.004** | **1.000–1.008** | **0.0284** | **↑ Significant** |
| IL-4 | **0.3817** | **0.1148–0.8997** | **<0.0001** | **↓ Significant** |
| IL-5 | 18.02 | 0.8856–641.2 | 0.0603 | ↑ Trend |
| IL-6 | 0.9999 | 0.9991–1.000 | 0.6813 | Not significant |
| EGF | 1.007 | 0.9935–1.027 | 0.3025 | Not significant |
| Eotaxin | 0.9850 | 0.9613–1.004 | 0.1306 | Not significant |

**Supplemental Table 2.** Logistic regression analysis of cytokine predictors of ASD diagnosis. Odds ratios (ORs), 95% confidence intervals (CIs), and p-values indicate the association between each cytokine and ASD diagnosis. ↑ indicates increased odds of ASD, whereas ↓ indicates decreased odds of ASD. Bold values indicate statistically significant associations (*p* < 0.05). IL-9, MCP-3, M-CSF,PDGF-AA, FGF-2, IL-1A, IL-1RA, and IL-4 were determined to be significantly associated with ASD diagnosis.
